# Fine-tuning the dose of recombinant human follicle-stimulating hormone alfa to individualize treatment in ovulation induction and ovarian stimulation cycles: a real-world database analysis

**DOI:** 10.3389/fendo.2023.1195632

**Published:** 2023-09-01

**Authors:** Anne E. Martini, Stephanie Beall, G David Ball, Brooke Hayward, Thomas D’Hooghe, Mary C. Mahony, Fabricio Collares, Allison B. Catherino

**Affiliations:** ^1^ National Institute of Child Health and Human Development, National Institutes of Health, Bethesda, MD, United States; ^2^ Reproductive Endocrinology and Infertility Department, Shady Grove Fertility Center, Rockville, MD, United States; ^3^ Seattle Reproductive Medicine Center, Seattle, WA, United States; ^4^ US Medical Affairs Fertility, EMD Serono, Inc., Rockland, MA, United States; ^5^ Global Medical Affairs Fertility, Research and Development, Merck Healthcare KGaA, Darmstadt, Germany; ^6^ Research Group Reproductive Medicine, Department of Development and Regeneration, Organ Systems, Group Biomedical Sciences, KU Leuven (University of Leuven), Leuven, Belgium; ^7^ Department of Obstetrics, Gynecology and Reproductive Sciences, Yale School of Medicine, New Haven, CT, United States; ^8^ Gynecologic and Obstetric Investigation, Basel, Switzerland; ^9^ Independent Scientific Affairs Consultant, Virginia Beach, VA, United States

**Keywords:** follitropin alfa, gonadotropin dose adjustments, individualized dosing, ovarian stimulation, ovulation induction, fertility treatment

## Abstract

**Introduction:**

Fine-tuning of injectable gonadotropin doses during ovulation induction (OI) or ovarian stimulation (OS) treatment cycles with the aim of using doses low enough to minimize the risk of excessive ovarian response while maintaining optimal efficacy may be facilitated by using an adjustable-dose pen injector. We examined the incidence and magnitude of individualized gonadotropin dose adjustments made during cycles of OI or OS, followed by either timed intercourse or intrauterine insemination, with or without oral medications, and assessed the relationship between patient characteristics and dosing changes using real-world evidence.

**Methods:**

This was an observational, retrospective cohort study using electronic medical records from a large US database of fertility centers. Data from patients who had undergone a first recombinant human follicle stimulating hormone alfa (r-hFSH-alfa/follitropin alfa) treated OI/OS cycle followed by timed intercourse or intrauterine insemination between 2015 and 2016 were included. Percentages of OI/OS cycles involving r-hFSH-alfa dose adjustments (in increments of ±12.5 IU or greater) with or without oral medications (clomiphene citrate or letrozole) were analyzed.

**Results:**

Of 2,832 OI/OS cycles involving r-hFSH-alfa administration, 74.6% included combination treatment with orals; 25.4% involved r-hFSH-alfa alone. As expected, the starting dose of r-hFSH-alfa was lower for cycles that used r-hFSH-alfa with orals than r-hFSH-alfa only cycles (mean [SD]: 74.2 [39.31] vs 139.3 [115.10] IU). Dose changes occurred in 13.7% of r-hFSH-alfa with orals versus 43.9% of r-hFSH-alfa only cycles. Dose adjustment magnitudes ranged from ±12.5 IU to ±450 IU. The smallest adjustment magnitudes (±12.5 IU and ±25 IU) were used frequently and more often for dose increases than for dose decreases. For r-hFSH-alfa with orals and r-hFSH-alfa only cycles, the smallest adjustments were used in 53.5% and 64.5% of cycles with dose increases and in 35.7% and 46.8% of cycles with dose decreases, respectively.

**Discussion:**

In OI/OS cycles followed by timed intercourse or intrauterine insemination, r-hFSH-alfa dose adjustments were frequent. In cycles that included orals, r-hFSH-alfa starting doses were lower and dose changes were fewer than with r-hFSH-alfa alone. Smaller dose adjustments facilitate individualized treatment with the goal of reducing the risks of multiple gestation, cycle cancellation, and ovarian hyperstimulation syndrome.

## Introduction

Ovulation induction (OI), a therapy that promotes the development of a single mature follicle and is followed by timed intercourse or intrauterine insemination (IUI), is widely used as a low-cost, less-invasive alternative to *in vitro* fertilization (IVF) for women with anovulatory subfertility or polycystic ovary syndrome (PCOS) (1–3). In contrast, ovarian stimulation (OS) is used for couples with unexplained infertility, endometriosis, or mild male factor infertility (1–5). OS aims to stimulate multiple mature follicles and can be followed by timed intercourse, IUI, or IVF. It should be noted that the analysis reported here was based on data collected prior to the 2017 harmonization of OI and OS definitions (6); therefore, data collection did not distinguish between OI and OS.

Currently, the balance of several randomized trials supports starting assisted reproduction with a more conservative treatment regimen of OS-IUI before moving to IVF for the treatment of unexplained infertility or mild male infertility in heterosexual couples (7). While OI/OS-IUI provides a less costly and less invasive alternative to IVF, multiple gestation rates and high risks of ovarian hyperstimulation syndrome are areas of concern. Independent of the stimulating drugs used, close monitoring of follicle development is considered of vital importance, and intracycle treatment adjustments might be required to avoid cycle cancellation or other undesired outcomes (7, 8).

Commonly used medications for OS or OI include oral agents such as antiestrogens (clomiphene citrate [CC]) or aromatase inhibitors (letrozole), and injectable gonadotropins (follicle stimulating hormone [FSH]), alone or in combination (3). CC is usually applied as first-line treatment for OI in anovulatory patients with PCOS, although letrozole may result in better pregnancy outcomes in this group (9). CC, available clinically since the early 1960s, is still frequently used for OS or OI although the mode of action has not been fully elucidated. Costs are low and side effects are mild, but at the expense of limited efficacy (3, 10) and increased multiple birth rates over the general population (10). Overall ovulation rates of ~60% to 70% with live birth rates of ~20% to 40% have been reported for women who started CC treatment (10). In patients with unexplained infertility, either CC or letrozole with IUI are considered to be similarly efficacious (3).

The use of injectable gonadotropins in OS or OI cycles and recombinant human follicle-stimulating hormone (r-hFSH-alfa), as well as their associated risk/benefit equations, are a consistent topic of controversy, particularly when discussing appropriate patient selection. The American Society for Reproductive Medicine do not recommend the use of gonadotropins for OS-IUI in unexplained fertility, alone or in combination with oral medications (3). However, in women with unexplained infertility, anovulatory subfertility, or PCOS, where oral agents are unsuccessful in inducing ovulation, the use of individualized low-dose injectable gonadotropins might represent a successful approach (11). In addition, a systematic review of randomized controlled trials comparing OS-IUI methods in couples with unexplained fertility reported that use of gonadotropins for OS increased the live birth rate compared to the oral OS medications CC or letrozole (12).

In October 2013, the first version of Gonal-f^®^ RFF Redi-ject^®^—a prefilled, preassembled, disposable pen injector—was registered in the US for the injection of follitropin alfa as a part of infertility treatment. An updated pen injector was approved by the FDA in 2017, featuring several elements designed to support prescribers and users, starting with a recommended minimal dose of 37.5 IU/mL with the ability to titrate between approved doses in ±12.5 IU increments. This new pen has undergone extensive testing to ensure it optimizes safety and efficacy for delivering multiple injections through a wide range of small dose adjustments, which is important for fine-tuning OS or OI and/or artificial reproductive technology (13).

Studies on OS for IVF have shown that, on more than one occasion during OS treatment with gonadotropins, the starting dose of recombinant human follicle-stimulating hormone (r-hFSH-alfa) had to be adjusted to meet the goal of optimizing outcomes while minimizing the risk of excessive ovarian response (1, 11). The appropriate starting dose for a particular patient can be determined based on clinical characteristics, including age, weight, relevant diagnoses, and ovarian reserve biomarkers (1, 14–16). Dosing may then be adjusted during the cycle or in subsequent cycles depending on patient response. The approach of individualizing gonadotropin doses in OS for IVF is common in clinical practice across the US and occurs more often in younger versus older patients (17), and has demonstrated comparable efficacy and increased safety compared with conventional fixed dosing (14). However, there is a lack of corresponding data on gonadotropin dose adjustments in OI/OS for timed intercourse or IUI.

Optimization of gonadotropin starting doses and dose adjustments occurred in 40.7% of cycles during OS for assisted reproductive technology, according to one report of real-world data (17), and in up to 45% of patients during OS for IVF, according to a recent systematic review of clinical studies (1). However, the prevalence and magnitude of dose adjustments in OI/OS for timed intercourse or IUI, and the clinical characteristics and demographics of patients undergoing OI/OS for timed intercourse or IUI with gonadotropins receiving dose adjustments have not, to the best of our knowledge, been assessed in real-world databases.

While convincing evidence of superiority of an individualized approach is expected to come from large, randomized trials comparing outcomes, the rationale for individualized dosing versus a one-size-fits-all strategy may need retrospective analyses of real-world data or large observational studies (18). In this context, real-world data from electronic medical records (EMRs) accumulated during routine clinical practice can provide valuable insight into clinical treatment practices.

This observational study assessed real-world data on r-hFSH-alfa dosing when included in the treatment regimen with and without oral agents. Treatment with a pen device allows dosing for OI/OS cycles to be fine-tuned with small dose-adjustment magnitudes. The goal of this analysis was to describe the real-world prevalence and magnitude of individualized gonadotropin dose adjustments (increases and/or decreases) made during OI/OS cycles in routine clinical practice, as well as to assess any association between patient characteristics and dosing changes.

## Materials and methods

This was a nonrandomized, observational, retrospective cohort study with secondary analyses of data from a large US database, including 39 fertility centers (IntegraMed America, Inc). As noted above, the database did not distinguish between OI and OS. The total dataset included 78,958 treatment-naïve patients whose initial treatment was categorized into three types: OI/OS with oral medications, with or without IUI; OI/OS with gonadotropins, with or without oral medication, and with or without IUI; or IVF. Initial treatment was OI/OS with gonadotropins, with or without oral medication, and with or without IUI, in 18,015 patients (22.8%). This subanalysis dataset included only data from patients who underwent their first r-hFSH-alfa-treated OI/OS cycle using an r-hFSH-alfa injection device from 2015 to 2016, with or without oral medication, and with or without IUI. Treatment cycles for IVF were not included. The 2015 start date for this subanalysis was selected to ensure that only patients using the updated device were included, as all earlier versions of the device had expired by this date. The updated r-hFSH-alfa injection device, Gonal-f^®^ RFF Redi-ject^®^ pen (follitropin alfa injection, EMD Serono, Inc., Rockland, MA, USA), allowed doses to be adjusted in ±12.5 IU increments when administered subcutaneously.

### Data source

The data were obtained from a large, real-world, EMR database and consisted of de-identified patient-level and cycle-level clinical and laboratory data for female patients who underwent fertility treatment in the US between January 1, 2015 and December 31, 2016. The data were collected from a network of 15 practices all using the same standardized EMR system; the practices comprised 39 clinics with 153 locations across the US and included patients from all 50 states. Ethics Committee/Institutional Review Board approval was not required as this analysis was based on data from a de-identified EMR database.

### Patient population

The dataset included 1,737 patients (or 2,832 treatment cycles) receiving r-hFSH-alfa administration. The analyses included treatment cycles identified as OI/OS, with or without the IUI component, which used subcutaneous gonadotropin injections, concomitant with or without oral medications (CC or letrozole).

Dose adjustment was defined as a change in r-hFSH-alfa dose after the start of OI/OS and was assessed during the treatment course, regardless of whether or how other medications were used in combination with r-hFSH-alfa during the same cycle. If r-hFSH-alfa treatment was combined with other drugs, dose adjustments of drugs other than r-hFSH-alfa were not considered. Patients’ baseline characteristics were analyzed and dosing characteristics per cycle were summarized by both daily dosing patterns and whether oral medications were used during OI/OS. The first cycle per patient was considered as the baseline; therefore, patient baseline characteristics were summarized by dosing pattern using their first cycle data only. For analysis of cycle-level dosing patterns, all cycles for a patient were included.

Data collected included age, antral follicle count (AFC), anti-Müllerian hormone (AMH) level, baseline diagnosis, Day 3 follicle stimulating hormone (FSH) levels for OI/OS cycles without oral medications, and treatment dose adjustments (in frequency and magnitude as ±IU).

### Statistical analyses

Continuous variables were summarized using descriptive statistics (number, mean, standard deviation [SD]). Categorical variables were summarized by number and percentages.

P-values were calculated using two-sample t-tests to compare starting dose and total dose of r-hFSH-alfa used in cycles that included orals versus cycles with r-hFSH-alfa alone. Due to the large sample size and to account for the multiple comparisons, all other P-values were considered significant at the two-sided α = 0.01 level. Analyses were completed using SAS software (version 9.4, Cary, North Carolina, USA).

## Results

A total of 2,832 treatment cycles involving r-hFSH-alfa administration (with or without oral medications) in 1,737 patients were included in the analysis.

Baseline data are presented by medications used (**Table 1**); 1,211 patients received r-hFSH-alfa with oral medications and 526 patients received r-hFSH-alfa without concomitant oral medication.

**Table 1:** Patient baseline characteristics stratified by use of oral medications with r-hFSH-alfa for OI/OS.

Patient characteristics	r-hFSH-alfa with orals			r-hFSH-alfa without orals		
	Total (n=1,211)	Constant dose (n=1,033)	Dose changes (n=178)	Total (n=526)	Constant dose (n=282)	Dose changes (n=244)
Age, years, mean (SD)	34.8 (4.9)	35.0 (4.8)	33.4 (4.9)	35.9 (5.0)	36.3 (4.9)	35.3 (5.2)
AFC, mean (SD)	16.1 (9.4)	15.6 (8.9)	19.1 (11.5)	16.6 (10.6)	15.4 (10.3)	18.5 (10.8)
AFC ≥12 (normal), n (%)	457 (37.7)	380 (36.8)	77 (43.3)	150 (28.5)	79 (28.0)	71 (29.1)
AMH, ng/mL, mean (SD)	3.6 (4.8)	3.2 (4.0)	5.8 (7.7)	3.6 (6.3)	2.5 (3.8)	4.9 (8.2)
AMH 1.5–4.0 ng/mL (normal), n (%)	340 (28.1)	303 (29.3)	37 (20.8)	86 (16.3)	44 (15.6)	42 (17.2)
AMH >4.0 ng/mL (high), n (%)	314 (25.9)	245 (23.7)	69 (38.8)	92 (17.5)	37 (13.1)	55 (22.5)
Primary Infertility Diagnosis*						
Ovulatory disorders/ PCOS, n (%)	182 (15.0)	133 (12.9)	49 (27.5)	91 (17.3)	35 (12.4)	56 (23.0)
Unexplained, n (%)	159 (13.1)	136 (13.2)	23 (12.9)	80 (15.2)	44 (15.6)	36 (14.8)
DOR, n (%)	142 (11.7)	125 (12.1)	17 (9.6)	137 (26.0)	87 (30.9)	50 (20.5)
Male infertility, n (%)	68 (5.6)	59 (5.7)	9 (5.1)	30 (5.7)	19 (6.7)	11 (4.5)
Endometriosis, n (%)	24 (2.0)	21 (2.0)	3 (1.7)	11 (2.1)	6 (2.1)	5 (2.0)
Nonhydrosalpinx tubal disease, n (%)	18 (1.5)	16 (1.5)	2 (1.1)	13 (2.5)	8 (2.8)	5 (2.0)
Uterine factor	11 (0.9)	8 (0.8)	3 (1.7)	2 (0.4)	2 (0.7)	0
Other/unknown, %	607 (50.1)	535 (51.8)	72 (40.4)	162 (30.8)	81 (28.7)	81 (33.2)

*The database used did not distinguish between OI and OS. However, based on current definitions, OI cycles took place in all women with ovulatory disorders/PCOS, and OS took place in all others.

AFC, antral follicle count; AMH, anti-Müllerian hormone; DOR, diminished ovarian reserve; OI, ovulation induction; OS, ovarian stimulation; PCOS, polycystic ovary syndrome; r-hFSH, recombinant human follicle-stimulating hormone; SD, standard deviation.

**Figure 1:**
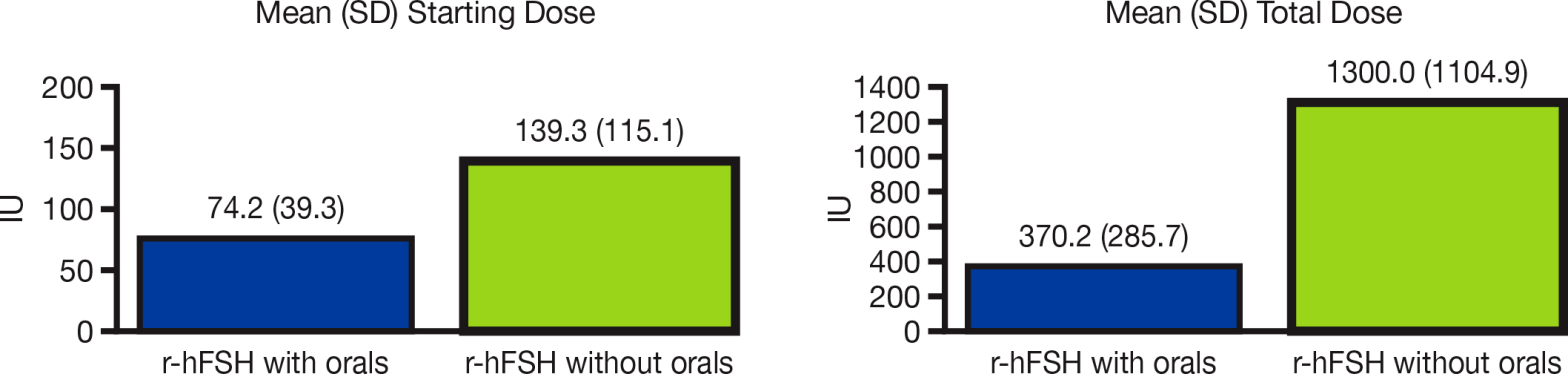
Mean r-hFSH-alfa starting dose and total dose in OI/OS cycles with and without oral medications. IU, international units; OI, ovulation induction; OS, ovarian stimulation; r-hFSH, recombinant human follicle-stimulating hormone; SD, standard deviation.

Of the total 2,832 OI/OS cycles that involved r-hFSH-alfa administration, 2,490 (87.9%) cycles used IUI. Combination treatment with orals was included in 2,112 (74.6%) cycles, whereas 720 (25.4%) used r-hFSH-alfa only. The (mean [SD]) starting dose of r-hFSH-alfa was lower for cycles that included orals (74.2 IU [39.3]) compared with r-hFSH-alfa only cycles (139.3 IU [115.1]) (p<0.0001, **Figure 1**). The (mean [SD]) total dose of r-hFSH-alfa was also lower for cycles that used r-hFSH-alfa with orals (370.2 IU [285.7]) compared with r-hFSH-alfa only cycles (1300.0 IU [1104.9]) (p<0.0001, **Figure 1**).

Patients in the r-hFSH-alfa with orals group had a lower mean age, were more likely to have a normal AFC, and were more likely to have normal or high AMH levels than those in the r-hFSH-alfa only group (**Table 1**). Patients in the r-hFSH-alfa with orals group were also less likely than those in the r-hFSH-alfa only group to have a primary infertility diagnosis of diminished ovarian reserve (DOR; 11.7% vs 26.0%), and more likely to have a primary infertility diagnosis of other/unknown (50.1% vs 30.8%).

### Dose adjustments

**Figure 2:**
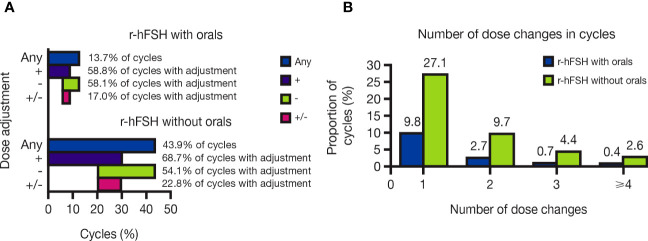
**(A)** Proportion of OI/OS cycles with and without oral medications that included r-hFSH-alfa dose changes and **(B)** Number of r-hFSH-alfa dose changes in OI/OS cycles with and without oral medications. +, cycles including a dose increase; -, cycles including a dose decrease; +/-, cycles including both a dose increase and a dose decrease; Any, cycles including any dose increase or decrease; OI, ovulation induction; OS, ovarian stimulation; r-hFSH, recombinant human follicle-stimulating hormone.

Overall, dose changes occurred in 13.7% of cycles with r-hFSH-alfa with orals versus 43.9% of r-hFSH-alfa–only cycles (**Figure 2A**). Dose adjustments were particularly common among patients with ovulatory disorders/PCOS who received r-hFSH-alfa without orals (occurring in 61.5% of these patients).

Of the cycles with dose changes, most included one dose change, with a single dose adjustment occurring in 9.8% and 27.1% of cycles with and without orals, respectively. Cycles with four or more dose adjustments were rare, comprising 0.4% and 2.6% of cycles with and without orals, respectively (**Figure 2B**). In cycles with dose adjustments, dose increases were slightly more common than decreases: increases occurred in 58.8% of dose-adjusted cycles with orals and 68.7% of those without orals, while decreases occurred in 58.1% of dose-adjusted cycles with orals and 54.1% of those without orals. Dose increases and decreases within the same cycle occurred in 17.0% and 22.8% of dose-adjusted cycles with and without orals, respectively (**Figure 2A**).

**Figure 3:**
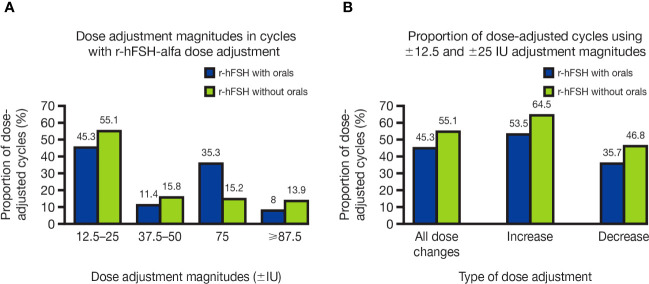
**(A)** Dose-adjustment magnitudes in OI/OS cycles with r-hFSH-alfa dose adjustment and **(B)** Proportion of OI/OS cycles with r-hFSH-alfa dose adjustment that used the smallest adjustment magnitudes (±12.5 and ±25 IU) for dose increases and decreases. **(A)** Where there were multiple dose changes in a single cycle, only the smallest dose-adjustment magnitude used is shown. **(B)** Cycles using ±12.5 and ±25 IU dose adjustments may have included multiple dose changes, i.e., included dose adjustments of higher magnitudes, as well as adjustments of ±12.5 and ±25 IU. IU, international units; OI, ovulation induction; OS, ovarian stimulation; r-hFSH, recombinant human follicle-stimulating hormone.

The magnitude of dose adjustments ranged from ±12.5 IU to ±450 IU. The smallest dose-adjustment magnitudes ( ±12.5 IU and ±25 IU) were used frequently: 45.3% of dose-adjusted cycles with orals and 55.1% of those without orals (**Figure 3A**). The smallest dose-adjustment magnitudes were used more often for dose increases (53.5% and 64.5% of dose-adjusted cycles with and without orals, respectively) than for dose decreases (35.7% and 46.8% of dose-adjusted cycles with and without orals, respectively; **Figure 3B**).

## Discussion

According to our findings, individualized r-hFSH-alfa dose adjustments were more prevalent during treatment cycles without oral medications than during treatment cycles with oral medications. In this real-world setting, dose adjustments were used in OI/OS cycles in patients with varying characteristics, and they were particularly common in patients with ovulatory disorders/PCOS.

These observations suggest that the use of small dose-adjustment magnitudes may facilitate individualized treatment, which could help in reducing risks for patients with more favorable prognoses (e.g., good ovarian reserves) undergoing a “low and slow” step-up OI/OS protocol for timed intercourse or IUI. Appropriate and individualized dosing during OI/OS for timed intercourse or IUI might help to mitigate risks such as those associated with ovarian multi-follicular response leading to multiple gestation or hyperstimulation syndrome (19), while preserving any advantage related to pregnancy rates (3).

In the present study, dose adjustments occurred in almost half of r-hFSH-alfa only cycles (43.9% of cycles included dose changes). In contrast, cycles with r-hFSH-alfa with orals had a more constant dosing approach (only 13.7% of cycles included dose changes). This finding is in line with clinical reasoning, as patients who received orals in addition to r-hFSH-alfa for OI/OS may have had a potentially better prognosis (i.e., better ovarian reserve and response) as they were more likely to be younger, have a normal AFC, and were less likely to have a primary diagnosis of DOR. Additionally, r-hFSH-alfa starting doses were lower and dose changes were fewer in OI/OS cycles that also included orals than in those who received r-hFSH-alfa only.

Our findings showed that dose adjustments were less common in patients with a diagnosis of unexplained infertility or DOR, compared with ovulatory disorders/PCOS. This could potentially be explained by a higher starting dose, reflecting a more aggressive initial treatment, which may not allow much flexibility for dose adjustments. Alternatively, it may be challenging to monitor OI/OS for dose adjustments in patients with poor ovarian response/reserve.

It is important to highlight that this analysis included only first treatment cycles with gonadotropins (with or without oral medications), independent of diagnosis, which reflected clinical practice at the time of data collection. Any cycle initiated/intended for IVF was not included in this analysis. For women with PCOS who are anovulatory with no other infertility factors, gonadotropins are recommended as second-line pharmacological agents to be used following failure of first-line oral therapy for OI (20). One must therefore consider why patients with PCOS in this dataset received gonadotropins in their first cycle. One potential explanation is that these patients could have undergone OI with oral medications before reaching a fertility clinic, for example, with a general obstetrician-gynecologist. Additionally, it has been recommended that gonadotropins could be considered as first-line treatment, with ultrasound monitoring, following counselling on cost and potential risk of multiple pregnancy, in women with PCOS who are anovulatory and have no other infertility factors (20).

In the present study, approximately two-thirds of patients with unexplained infertility who underwent a first treatment cycle with r-hFSH-alfa received concomitant oral medications, while one-third received r-hFSH-alfa only. A systematic review of randomized controlled trials comparing OS-IUI methods in couples with unexplained fertility reported that use of gonadotropins for OS increased the live birth rate compared to the oral OS medications CC or letrozole (12). Treatment paradigms for unexplained infertility have typically involved OS with oral medications, then OS with gonadotropins, followed by IVF for those unsuccessful in achieving pregnancy with OS (3). It should be remembered that this analysis is based on real-world data collected from an existing database and represents clinical practice from previous years.

Dose adjustments in OS or OI cycles, whether for IUI or IVF, aim to induce sufficient ovarian response to better obtain optimal outcomes for live birth, while avoiding the risks of multifollicular ovulation, cycle cancellation, and/or ovarian hyperstimulation syndrome (8, 14, 19). Achieving the balance between the desired efficacy and necessary safety may require small dose increases, decreases, or both based on careful monitoring of ovarian response (12, 21, 22).

The present study found that dose increases were slightly more common than decreases, particularly in cycles with r-hFSH-alfa only, and a subset of cycles included both increases and decreases. Where cycles included both an increase and decrease, this was likely due to concerns about potential side effects of an increased dose. Although dose-adjustment magnitudes ranged from ±12.5 IU to ±450 IU, the smallest magnitudes ( ±12.5 IU and ±25 IU) were used in approximately half of the dose adjustments recorded, both for cycles with orals and those with r-hFSH-alfa only. The assessment of small dose-adjustment magnitudes was possible because of the capabilities of the updated Gonal-f^®^ RFF Redi-ject^®^.

The capability of the Gonal-f^®^ RFF Redi-ject^®^ to make small-magnitude dose-adjustments is in line with the American Society of Reproductive Medicine Practice Committee opinion from 2020 (19), where the recommended approach for gonadotropin regimens for OI in anovulatory women is to begin with a low dose of gonadotropin—typically 37.5–75 IU/day— in the first dose-finding cycle and increase the dose in small increments after seven days or more if no follicle >10 mm in size has developed. In subsequent cycles, treatment generally begins at the threshold of response that has been previously determined. Although 7–12 total days of treatment is typical, longer durations of treatment may be required. Once a mature follicle has developed, exogenous human chorionic gonadotropin is administered to stimulate ovulation.

The limitations of this study include a relatively small sample size and the descriptive nature of the study design, which did not include clinical outcomes that could help to understand the impact of the observed dosing changes. Other data that were not accessible for analysis in the database include the length of r-hFSH-alfa treatment and the starting day of r-hFSH-alfa in the overall treatment cycle for OI/OS cycles with orals. Nonetheless, this work reflects clinical practice and can be used to complement the results from randomized controlled studies. As much as possible, the combination of data from large observational studies and data from prospective controlled trials are still necessary to identify the optimal regimen of stimulation for OI/OS cycles.

In conclusion, in OI/OS cycles, r-hFSH-alfa dose adjustments in a real-world setting are frequent. In cycles treated with r-hFSH-alfa concomitant with oral medication (CC or letrozole), r-hFSH-alfa starting and total doses were lower and dose changes were fewer than with r-hFSH-alfa alone. Smaller dose-adjustment magnitudes facilitate individualized treatment with the goal of reducing the risks of multiple gestation, cycle cancellation, and ovarian hyperstimulation syndrome.

## Data availability statement

The data analyzed in this study is subject to the following licenses/restrictions: The proprietary database used for this study was made available to EMD Serono, Inc., Rockland, MA, USA through a license that limits dissemination of the data, thus they have not been made publicly available. Requests to access these datasets should be directed to Krys Modrzejewski, krys.modrzejewski@emdserono.com.

## Ethics statement

Ethical approval was not required for the study involving humans in accordance with the local legislation and institutional requirements. Written informed consent to participate in this study was not required from the participants or the participants’ legal guardians/next of kin in accordance with the national legislation and the institutional requirements.

## Author contributions

MM, TD’H, BH, AC, SB, FC, and AM conceived and designed the study. AM, SB, and GB collected the data. BH conducted the analyses. All authors contributed to the article and approved the submitted version.
